# Pattern of Contrast Sensitivity Changes in Acute Central Serous Chorioretinopathy

**DOI:** 10.1155/2017/9053932

**Published:** 2017-12-20

**Authors:** Preeyachan Lourthai, Patama Bhurayanontachai

**Affiliations:** Department of Ophthalmology, Faculty of Medicine, Prince of Songkla University, Songkhla 90110, Thailand

## Abstract

**Purpose:**

To evaluate contrast sensitivity (CS) changes in acute central serous chorioretinopathy (CSC).

**Methods:**

Visual acuity (VA), CS, and subretinal fluid (SRF) were evaluated monthly for 6 months. Treatment was considered at 3 months in case of persistent SRF.

**Results:**

Twelve of 20 eyes (60%) had spontaneous SRF resolution within 4 months. Five of 8 patients with delayed SRF resolution received either focal laser or photodynamic therapy. The CS was impaired in all spatial frequencies at baseline. There was a negative correlation between the baseline SRF thickness and CS at 3 and 6 cycles per degree (cpd). The CS improved significantly at the time of fluid resolution (*p* = 0.001) and continued to improve in 3 and 6 cpd. The CS at 6 cpd did not recover if compared to a normal fellow eye at 6 months (*p* = 0.018). The CS of 12 cpd at 6 months was superior in the spontaneous resolution group.

**Conclusion:**

The impaired CS gradually improved as the SRF reduced at all spatial frequencies. CS at 3 and 12 cpd continued to improve after complete fluid resolution. Despite an excellent final VA, the CS at 6 months did not regain its normal value.

## 1. Introduction

Central serous chorioretinopathy (CSC) is an idiopathic disease of the retinal pigment epithelium (RPE) and choroid characterized by a focal well-circumscribed, serous detachment of the neuroepithelium in the macular region. CSC affects predominantly healthy men aged between 25 and 55 years. Patients may experience a variety of visual disturbances from asymptomatic to a sudden onset of blurred vision, micropsia, metamorphopsia, dyschromatopsia, or paracentral scotoma [1]. The visual acuity (VA) varies, but it is frequently better than 20/30. Due to a subtle disturbance in VA, some studies suggested other means to evaluate the visual function for early diagnosis of CSC, including contrast sensitivity (CS) [2] and microperimetry [3]. The CS test, which measures both spatial frequency and CS, has superior reflection on real-life visual capability such as reading, face recognition, and driving [4].

The CSC is usually self-limited with a good visual prognosis. The subretinal fluid (SRF) resolves spontaneously with an average resolution time of 3-4 months in 80–90% of patients. Most of the eyes achieve their initial VA after resolution in the acute stage. In chronic cases with persistent SRF, visual function deterioration is due to RPE and photoreceptor damage. Laser photocoagulation or photodynamic therapy (PDT) is indicated in chronic cases. The procedures can induce rapid fluid resorption within several weeks of therapy; however, the benefit on visual outcome and recurrence rate are controversial [5]. After fluid resolution in the acute stage, some patients still complain of mild residual visual disturbances. Diminished CS is a common sequela of CSC which affects a patient's daily tasks such as reading [6–9].

Most of the previous studies assessed visual outcome based on a routine VA test [5, 10–12]. There is limited evidence on CS during the acute phase (<3 months). This study aims to evaluate VA as well as CS during and after the acute stage of CSC either with or without intervention. The study provides insights on the natural history of the disease and the effects of SRF on CS which will be beneficial for an early diagnosis, follow-up guideline, and treatment of choice in the future.

## 2. Patients and Methods

### 2.1. Participants

Twenty patients diagnosed with acute CSC were recruited from Songklanagarind Hospital between April 2014 and March 2015. The inclusion criteria were onset of CSC within the previous 2 months, age ≤ 50 years, and a fundus fluorescein angiogram (FA) that confirmed the diagnosis of active CSC. The exclusion criteria were recurrent acute CSC with poor VA, previous acute CSC in the fellow eye with subnormal visual outcome, history of other retinal disease, significant ocular media opacity with possible progression within 6 months, previous ocular surgery within 6 months, previous macular laser treatment, steroid use, pregnancy, and allergy to fluorescein dye. The sample size of this study was based on previous prospective studies of CSC [6, 9, 13]. The study protocol was reviewed and approved by the Ethics Committee of the Faculty of Medicine, Prince of Songkla University. The study was conducted in compliance with the Declaration of Helsinki. Informed consents were signed by the patients after the protocol was thoroughly explained.

### 2.2. Study Design

**Figure 1:**
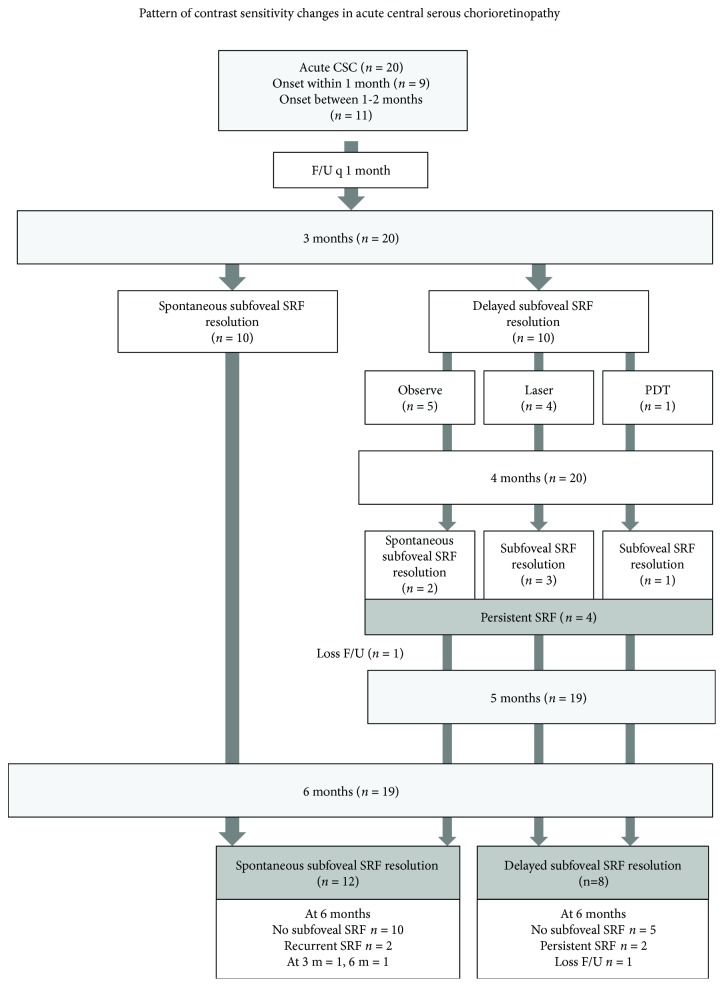
Flow of the patients in the study.

This study was a prospective observational study. The flow chart of the study is shown in Figure 1. The patients were evaluated at baseline and monthly until 3 months. Spontaneous resolution of SRF was determined at 3 months. Patients with spontaneous resolution of SRF were reevaluated at the 6-month end-point. Patients with persistent SRF underwent FA and were considered for appropriate treatment based on the investigator's decision and then continued their monthly follow-ups until the 6-month end-point.

### 2.3. Treatment for CSC

Songklanagarind Hospital has a standard treatment protocol for CSC patients. Extrafoveal leakage is treated by subthreshold laser therapy. A 532 nm green laser (spot size 100 *μ*m, duration 0.05 seconds, and power 50 mW) is applied at the leakage site and titrated up to achieve a faint grey spot on the retina. Subfoveal or juxtafoveal leakage is treated by half-dose PDT [11] with 3 mg/m^2^ of verteporfin (Visudyne®, Novartis Pharma, Basel, Switzerland) that is infused over 7 minutes followed by delivery of a laser at 689 nm for 10 minutes from the commencement of infusion to the target leakage area. A total light energy of 50 J/cm^2^ is achieved by delivering a 600 mW/cm^2^ light intensity over 83 seconds. The laser spot size is determined by a diameter of 1000 *μ*m larger than the greatest linear dimension of the leaking point.

### 2.4. Data Collection

The demographic data were recorded (Table 1). At each visit, all patients underwent history taking and complete ophthalmic examination of both eyes through dilated pupils under slit lamp biomicroscopy and spectral-domain optical coherence tomography (SD-OCT) (Spectralis®, Heidelberg Engineering, CA, USA). Fluorescein angiography (FA) (Heidelberg Spectralis OCT®, Heidelberg Engineering, Heidelberg, Germany) was performed only at baseline for the diagnosis of all patients. An additional FA was considered at the third month to identify a leaking point if persistent SRF remained.

At each visit, the best-corrected VA (BCVA) and CS were measured before pupil dilatation. The BCVA was measured at a 4-meter distance by an Early Treatment Diabetic Retinopathy Study chart and was converted to a logarithm of minimum angle of resolution (logMAR) value. The CS was tested with best correction using the CSV-1000 grating charts (VectorVision® Inc., Greenville, OH, USA) under photopic conditions. The value was recorded in log scale.

The SRF and choroidal thickness values from OCT were measured manually using the caliper provided in the Heidelberg Spectralis-OCT software. The SRF thickness was defined as a distance between the inner surface of the RPE and the outer surface of the neurosensory retina at the subfoveal region. The choroidal thickness, using the enhanced depth imaging SD-OCT technique to assist in visualization of the choroid [14], was measured under enhancement from the outer surface of the line formed by the RPE to the inner surface of the observed sclera at the subfoveal region.

### 2.5. Statistical Analysis

The descriptive analysis included mean ± standard error or median (range) that depended on the data distribution. The statistical analysis was done using the Pearson *χ*^2^ test, Mann–Whitney test, Wilcoxon signed-rank test, and Pearson correlation. A *p* ≤ 0.05 was considered statistically significant.

## 3. Results

### 3.1. Demographic and Clinical Characteristics

Twenty eyes of 20 patients diagnosed with CSC were recruited in the study. The demographic data of the patients at the initial examination are listed in Table 1. The mean age was 39.85 ± 5.59 years (range 29–48 years). Nine patients (45%) had an onset of CSC within the previous 1 month, and 11 patients (55%) had an onset of CSC within the previous 1 to 2 months. In one patient with bilateral involvement, only the symptomatic eye was recruited.

### 3.2. Clinical Courses

Figure 1 shows the flow of the patients in the study. Nineteen patients (95%) completed the 6-month follow-up. One patient was lost to follow-up at month 5. Spontaneous SRF resolution was observed in 12 patients (60%): at 1 month (*n* = 2); 2 months (*n* = 2); 3 months (*n* = 6); and 4 months (*n* = 2). Delayed SRF resolution at 3 months was noted in 10 patients. Two patients had minimal remaining SRF at 3 months; therefore, the treatment was postponed for another month. These two patients eventually developed a complete resolution of SRF at 4 months and were considered to be in the spontaneous SRF resolution group. The remaining eight patients with persistent SRF were considered for treatment that consisted of focal laser (*n* = 4), half-dose PDT (*n* = 1), or observe (*n* = 3). Posttreatment resolution was achieved within 1 month in a patient who received PDT and in three out of four patients who received laser treatment. One out of three patients in the observe group eventually completed resolution at 6 months, one was lost to follow-up, and only one had persistent SRF throughout the study. Two patients in the spontaneous group developed a recurrence, one at month 3 and another at month 6. The presence of SRF at 6 months was the result of persistent SRF in 2 patients and recurrence of CSC in 2 patients.

### 3.3. Resolution of Subretinal Fluid

**Figure 2:**
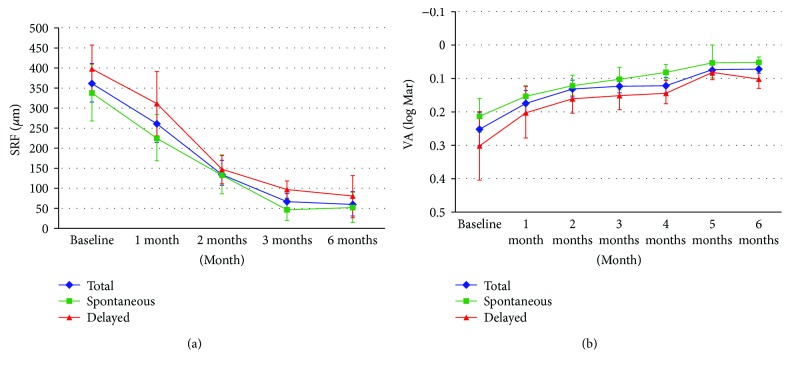
(a) Changes in mean subfoveal subretinal fluid thickness (*μ*m) and (b) changes in mean visual acuity (logMAR) in overall, spontaneous, and delayed subfoveal fluid resolution groups.

In a comparison between the spontaneous and delayed resolution groups, the baseline SRF thicknesses were similar (*p* = 0.270). The overall rate of SRF thickness resolution was rapid during the first 3 months and decelerated thereafter (Figure 2(a)). At 3 months, the SRF thickness was higher in the delayed resolution group than in the spontaneous resolution group (96.38 ± 62.88 *μ*m versus 46.75 ± 95.7 *μ*m, *p* = 0.047). At the endpoint of the study, the SRF thickness was similar between the two groups (51.42 ± 130.48 *μ*m in the spontaneous group versus 79.57 ± 140.29 *μ*m in the delayed group, *p* = 0.773).

### 3.4. Visual Acuity Change

The overall BCVA change is shown in Figure 2(b). The baseline mean of BCVA (0.25 ± 0.23 logMAR) gradually improved over 6 months regardless of the rate of SRF resolution (0.12 ± 0.12 logMAR at 3 months and 0.07 ± 0.06 logMAR at 6 months). At the time of SRF resolution and at 6 months, the overall BCVA had significantly recovered.

At 6 months, all of the patients attained BCVA in the 20/20–20/32 range (20/20 *n* = 8; 20/25 *n* = 10; 20/32 *n* = 1). Three patients with persistent SRF at 6 months had BCVA in the 20/25–20/32 range. Considering only the patients without subfoveal SRF, all of them had BCVA not worse than 20/25 (*n* = 16). Between the spontaneous resolution group and delayed resolution group, the mean BCVA at baseline, 3 months, and 6 months was not statistically significant.

### 3.5. Contrast Sensitivity in Acute CSC

**Figure 3:**
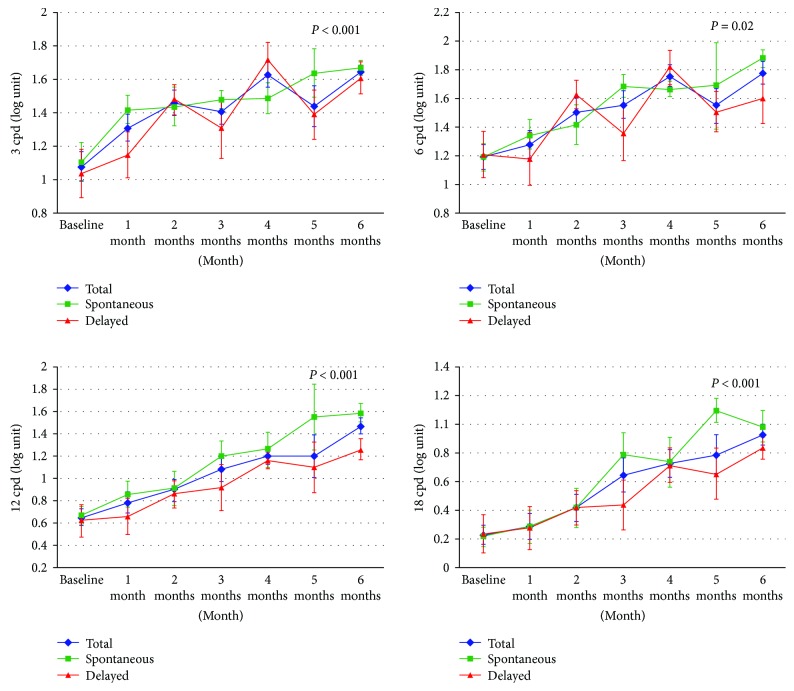
Serial changes in contrast sensitivity at 3, 6, 12, and 18 cycles per degree (cpd). Each line represents the overall patients, spontaneous, and delayed subfoveal fluid resolution groups.

Figure 3 shows the CS at each time point at 4 different spatial frequencies (3, 6, 12, and 18 cycles per degree (cpd)). The mean CSs at baseline were 1.08 ± 0.39, 1.19 ± 0.38, 0.65 ± 0.33, and 0.23 ± 0.30 at 3, 6, 12, and 18 cpd, respectively. Overall, the patients had gradual improvement of CS at all frequencies over 6 months. There was a significant negative correlation between the subfoveal SRF thickness and 3 cpd and 6 cpd at baseline (*R* = −0.518 and 0.573; *p* = 0.019 and *p* = 0.008, resp.). At the time of complete SRF resolution, the CS improved significantly in all spatial frequencies (*p* = 0.001). The improvement was more distinct at higher spatial frequencies. After the SRF had completely reabsorbed (quiescent phase), the CS continued to improve until 6 months. The improvement of CS at the quiescent phase was more pronounced at 3 and 12 cpd (*p* = 0.015 and 0.020, consecutively).

In a comparison between the affected eyes with the normal fellow eyes at 6 months (*n* = 12, excluded fellow eyes with current or previous CSC), only the CS at 6 cpd of the patients with complete SRF resolution was significantly lower than the normal fellow eye (*p* = 0.018, *n* = 12). A subgroup analysis found that this reduction in CS at 6 cpd was significant in only the delayed resolution group (*p* = 0.043), not in the spontaneous resolution group (*p* = 0.593).

A comparison between the spontaneous and delayed resolution groups revealed that the CS values at baseline and at the time of SRF resolution were similar at all spatial frequencies. At 6 months, the CS at 12 cpd in the spontaneous resolution group was significantly superior to the CS of the delayed resolution group (1.59 ± 0.28 versus 1.26 ± 0.25, *p* = 0.017). The CS value at other spatial frequencies was not statistically different.

### 3.6. Choroidal Thickness Changes

The overall average choroidal thickness at baseline was 397.55 ± 80.81 *μ*m and was similar in both spontaneous and delayed resolution groups. The mean choroidal thickness at 6 months was 371.63 ± 72.11 *μ*m, which had significantly decreased by 22.88 ± 24.26 *μ*m (*p* = 0.004) compared to the baseline. There was no statistical difference between the spontaneous and delayed groups (*p* = 0.536).

## 4. Discussion

This study examined the course of CS in acute CSC patients during a 6-month follow-up period [15]. The demographic data, risk factors, and clinical characteristics of the recruited patients at baseline were comparable to previous series of CSC [12, 16–18]. In our study, the history of stress (50%) and current smoking (45%) was common. A complete resolution of subfoveal SRF could be found as early as 1 month from the onset. The majority of the patients had a resolution of subfoveal fluid within 3 months after the onset which was similar to previous literature reports [1]. Two patients with minimal SRF at month 3 eventually had spontaneous resolution at month 4. This result suggested that the SRF could be observed up to 4 months in selected cases. However, persistent SRF in patients beyond 4 months rarely self-resolves; therefore, intervention is needed in these patients. The recurrence rate at 6 months was 10% in our study, which was lower than previous reports (15.4–50.7%). This can be explained by a longer follow-up time in the previous series [12].

The mean BCVA for the affected eye in this study was also comparable to other series (20/25–20/40). Three patients with marked impaired baseline BCVA (20/80–20/125) were found to have a higher SRF thickness than the other patients. The BCVA gradually improved over time as with the reduction of the SRF thickness, and it continued to improve even in the quiescent phase. At 6 months, all of the patients without subfoveal SRF achieved excellent BCVA; however, the BCVA could be compromised if the SRF persisted. There was no statistical difference in the BCVA at 6 months between the spontaneous and delayed resolution groups. Despite a relatively good BCVA outcome at 6 months, some patients still complained of visual disturbance.

Our study showed significantly compromised CS at baseline with a negative correlative between the baseline subfoveal SRF thickness and CS at 3 and 6 cpd. The CS gradually improved during the course of the disease as the subfoveal SRF reduced and was significantly enhanced at the time of complete SRF resolution in all spatial frequencies. The presence of SRF at any time point noticeably impaired the CS. In the quiescent phase, the CS at 3 and 12 cpd continued to improve until the end of the study.

At 6 months, despite a complete SRF resolution, only the CS at 6 cpd did not regain its normal range as in the unaffected fellow eye. Furthermore, the overall CS after resolution showed lower values compared to the normative data of CS in Caucasian subjects [19]. A previous study proposed the hypothesis that the fellow eye of CSC could have a subclinical manifestation [7]. The CS at 3 and 18 cpd in the fellow eye of CSC was compromised compared to the age-matched normal eye. Therefore, CS of the fellow eye might not represent a true normal CS value for the study. The CS values at 12 and 18 cpd were affected more than those at 3 and 6 cpd. This corresponded to a previous study that the CS at high and intermediate spatial frequencies was impaired in acute CSC and significantly correlated with VA [13]. Only at 12 cpd was the CS significantly superior in the spontaneous resolution group compared to the delayed resolution group.

This study showed that the subfoveal choroidal thickness also increased in the affected eye, compared to the normal value of 287 ± 76 *μ*m in previous reports [20, 21]. This finding corresponds to the previous studies that showed a choroidal thickening in CSC [22–24]. Our study found that the choroidal thickness gradually reduced over 6 months. On the contrary, Brandl et al. [25] reported that significant thickness reduction occurred at 1 week follow-up. This difference might be from an application of nonsteroidal anti-inflammatory and brinzolamide eye drops accompanied with oral acetazolamide in their study.

A CS test reflects better daily activities and provides a useful tool for clinical evaluation during the course of the disease. Recovery of the BCVA and CS began as soon as the process of SRF resolution and continued even in the quiescent phase. After an episode of CSC, the BCVA can recover to normal, but not the CS. An improvement of CS values between the spontaneous and delayed SRF resolution was not significantly different, except for CS at a spatial frequency of 12 cpd. A recent study proposed early treatment to minimize irreversible photoreceptor damage from SRF accumulation, thus resulting in better VA and CS in the higher spatial frequencies [5].

A limitation of this study is the observational prospective study design. Furthermore, the study was unable to control some confounding factors and the sample size was small. A study with a larger sample size is needed for more statistically significant data. A longer follow-up period may also give better information on the long-term outcome.

In conclusion, the CS was significantly impaired in acute CSC. During the course of the disease, the recovery of BCVA and CS began as soon as resorption of the SRF had begun and continued to recover after complete fluid resolution. Despite recovery of the BCVA to a normal level, CS only partially recovered. High spatial frequencies were mainly affected. The delay in SRF resolution resulted in a compromised CS outcome. Hence, CS might be useful in the evaluation of patients with CSC.

## Figures and Tables

**Table 1:** Demographic and clinical characteristics at baseline.

Parameter	Total *n* = 20	Spontaneous resolution *n* = 12 (60%)	Delayed resolution *n* = 8 (40%)	*p* value
Gender				
Male	17 (85)	10 (83.3)	7 (87.5)	1.000
Female	3 (15)	2 (16.7)	1 (12.5)	—
Age, years, mean ± SD	39.85 ± 5.59	40.92 ± 6.11	38.25 ± 4.62	0.434
Ocular history				
Other previous ocular disease	1 (5)	1 (8.3)	0 (0)	1.000
Recurrent ipsilateral eye	3 (15)	3 (25.0)	0 (0)	0.242
History in fellow eye	4 (20)	3 (25.0)	1 (12.5)	0.619
Systemic history				
DM	0 (0)	0 (0)	0 (0)	—
HT	0 (0)	0 (0)	0 (0)	—
DLP	3 (15)	3 (25.0)	0 (0)	0.242
GERD	5 (25)	2 (16.7)	3 (37.5)	0.347
Others	6 (30)	4 (33.3)	2 (25.0)	1.000
Smoker	10 (50)	5 (41.7)	5 (62.5)	0.650
Stress	9 (45)	6 (50)	3 (37.5)	0.670
Days of onset, mean ± SD	21.10 ± 16.58	20.58 ± 17.39	21.88 ± 16.45	0.581
Laterality				
RE	11 (55)	6 (50.0)	5 (62.5)	0.303
LE	8 (40)	6 (5.0)	2 (25.0)	—
BE	1 (5)	0 (0)	1 (12.5)	—
Symptom				
Metamorphopsia	4 (20)	3 (25)	1 (12.5)	0.619
Central scotoma	15 (75)	10 (83.3)	5 (62.5)	0.347
Generalized blurring	3 (15)	1 (8.3)	2 (25.0)	0.537
BCVA				
LogMAR, median (min, max)	0.17 (0, 0.82)	0.17 (0, 0.7)	0.16 (0, 0.82)	0.910
20/20–20/32	14 (70)	9 (75)	5 (62.5)	0.591
20/40–20/50	3 (15)	2 (16.7)	1 (12.5)	—
<20/50	3 (15)	1 (8.3)	2 (25)	—
Spherical equivalent, diopter, median (min, max)	0.31 (−1.0, 1.25)	0.38 (0.5, 1.13)	0.13 (−1.0, 1.13)	0.624
FA pattern of leakage				
Inkblot	16 (80)	9 (75.0)	7 (87.5)	0.680
Smokestack	2 (10)	1 (8.3)	1 (12.5)	—
Combined	1 (5)	1 (8.3)	0 (0)	—
No leakage	1 (5)	1 (8.3)	0 (0)	—
SRF thickness (*μ*m), mean ± SD	362.40 ± 213.63	338.42 ± 244.56	398.38 ± 165.61	0.270
Choroidal thickness (*μ*m), mean ± SD	397.55 ± 80.81	381.67 ± 84.89	421.38 ± 72.95	0.427
Presence of PED	10 (50)	6 (50)	4 (50)	1.000

Data are presented as *n* (%) unless indicated otherwise. SD: standard deviation; DM, diabetes mellitus; HT: essential hypertension; DLP: dyslipidemia; GERD: gastroesophageal reflux disease; RE: right eye; LE: left eye; BE: both eyes; BCVA: best-corrected visual acuity; FA: fluorescein angiogram; SRF: subretinal fluid; PED: pigmented epithelial detachment.
